# Stimuli-responsive fluorescence switching of cyanostilbene derivatives: ultrasensitive water, acidochromism and mechanochromism†

**DOI:** 10.1039/c8ra03598a

**Published:** 2018-06-21

**Authors:** Bin Wang, ChunYing Wei

**Affiliations:** Key Laboratory of Chemical Biology and Molecular Engineering of Ministry of Education, Institute of Molecular Science, Shanxi University Taiyuan 030006 PR China weichuny@sxu.edu.cn; School of Basic Medical Sciences, Shanxi Medical University Taiyuan 030001 P. R. China (+)86-351-7010699

## Abstract

A novel donor–π–acceptor structure stimuli-responsive fluorescent material of (*Z*)-2-(4′-(diphenylamino)-[1,1′-biphenyl]-4-yl)-3-(pyridin-2-yl)acrylonitrile (*o*N-TPA) was designed and synthesized, with the cyano-group and pyridine as the acceptors (A) and triphenylamine as the donor (D). *o*N-TPA exhibits an obvious solvatochromic effect and the excited state is confirmed to be a hybridized local and charge-transfer (HLCT) state that simultaneously possesses the locally-excited (LE) state and charge transfer (CT) state characters. The LE state ensures relatively high fluorescence efficiency while the CT state provides multi-stimuli responsive fluorescence behaviors because it is easily tuned by the surrounding environment. Firstly, *o*N-TPA exhibits “on–off–on” fluorescence properties in the mixture of water/tetrahydrofuran (THF) with the increasing water content. For the “on–off” part, a good linear relationship between fluorescence intensity and water fraction is achieved, which is ascribed to the synergistic effect of protons in water and intramolecular charge-transfer (ICT) effect depending on solvent polarity. The “off–on” part demonstrates the aggregation-induced enhanced emission (AIEE) character of *o*N-TPA. Secondly, *o*N-TPA can be used as a protonic acid sensor to detect trifluoroacetic acid (TFA) in solvent and HCl vapour in the solid state due to the binding of the proton to the pyridine group. Finally, *o*N-TPA presents remarkable and reversible mechanochromic fluorescence switching between 552 nm and 642 nm (90 nm red-shift) during the pressurizing–depressurizing process. This work not only comprehensively demonstrates the stimuli-responsive fluorescence behaviors of *o*N-TPA, but also provides a D–π–A structure fluorescent material possessing potential applications in detection and sensing with remarkable fluorescence changes.

## Abstract 2

A donor–acceptor dye exhibits high luminescence efficiency and high-contrast piezochromism with HLCT properties. The doped films show the ratiometric photo-luminescence peak shift under high pressure, interestingly, making them piezo-sensors.

## Graphical abstract

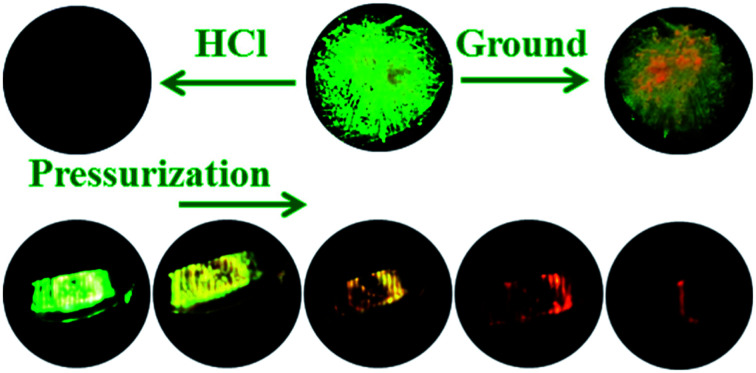

## 1. Introduction

Stimuli-responsive fluorescent materials have recently attracted more and more attention due to their potential applications as sensors, switches, and recording media in the past few years.^1^ Recently, various organic materials with efficient and reversible fluorescence behaviors have been developed, responding to light,^2^ mechanical force,^3^ temperature,^4^ pH,^5^ water,^6^ and solvent vapors.^7^ In the design of stimuli-responsive materials, a high solid-state efficiency is necessary for achieving obvious changes in fluorescence behavior towards various external stimuli. However, most luminescent materials suffer from the effects of aggregation-caused quenching (ACQ), which limits the application of chromic materials. A phenomenon named aggregation-induced enhanced emission (AIEE) appeared in 2001, which demonstrated enhanced emission in the aggregate state for the restriction of intramolecular rotations (RIR).^8^ From then on, more and more AIEE character luminescent materials have been developed and studied in organic light-emitting diodes (OLEDs),^9^ luminescent sensors,^10^ biomedical imaging,^11^ and smart materials.^12^ Except for the typical AIEE group of tetraphenylethene (TPE), many other systems have also been found to possess AIEE character.^13^

Charge transfer (CT) fluorescent complexes usually consist of electron donor (D) and electron acceptor (A) parts, which have been extensively exploited in OLEDs,^14^ organic photo-voltaics (OPVs),^15^ field-effect transistors (FETs),^16^ nonlinear optics, and fluorescent chemosensors.^17^ CT molecules always show the strong solvatochromic effect, and their photoluminescence (PL) emissions exhibit a large red shift with the increase of solvent polarity. Therefore, the fluorescence emissions of CT-materials are greatly dependent on their excited states, which can be easily tuned by the surrounding environment, such as external stimulus. However, the strong CT-state usually causes a serious decrease in PL efficiency due to the small orbital overlap. Ma *et al.* proposed a named hybridized local and charge-transfer (HLCT)^18^ state method to realize high fluorescence efficiency (*η*_PL_), and this excited state simultaneously possesses the locally-excited (LE) state and CT state characters. The LE component ensures relatively high fluorescence efficiency, and the CT component provides multi-stimuli responsive fluorescent behavior, which may provide a new strategy to achieve high-efficient and high-contrast stimuli responsive materials.

**Scheme 1:**
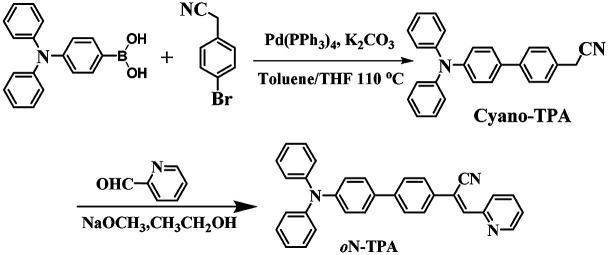
Synthetic route to *o*N-TPA.

Herein, we report a D–π–A structure molecule of (*Z*)-2-(4′-(diphenylamino)-[1,1′-biphenyl]-4-yl)-3-(pyridin-2-yl)-acrylonitrile (*o*N-TPA) (Scheme 1), which exhibits the multi-stimuli responsive fluorescent properties. Solvatochromic experiments exhibit a large red-shift from 468 nm in low polar hexane to 615 nm in high polar acetonitrile. Combined with theoretical calculation and Lippert–Mataga model,^19^ the excited state is confirmed to be a HLCT state. The photophysical properties of *o*N-TPA in the mixture of THF/water demonstrate a pronounced AIEE effect. Moreover, it shows ultra-sensitivity PL response to water in THF, which can be used as a detector for water in organic solvents. The color and intensity of fluorescence produce the substantial changes by protonation, thus enabling it to work as a fluorescent pH sensor in both solution and solid states. At the same time, the switchable fluorescence of *o*N-TPA in the solid state is achieved during pressurizing–depressurizing process. Therefore, this work provides a comprehensive insight into the stimuli-responsive fluorescence behavior of a HLCT state luminescent material and a new strategy to achieve high efficient and easily tuned stimuli-responsive materials.

## 2. Experimental section

### 2.1 Constant-pressure experiments

The ^1^H NMR spectra were recorded by a Bruker Avance III 500 MHz spectrometer with DMSO-*d*_6_ as solvent and tetramethylsilane (TMS) as standard. The Shimadzu UV-2600 spectrophotometer (Japan) and SENS-9000 (GildenPhotonics, England) spectro-photometer were used to record UV-vis and PL spectra, respectively, and a solution of the sample (*ca.* 10^−5^ M) in a 1 cm quartz cuvette was used in the measurement. Digital photographs were taken by 550D (Canon, Japan) digital cameras. We used spectrometer C11347 (Hamamatsu, Japan) to measure their absolute fluorescence quantum yields. Powder XRD measurements were conducted in the range 5 < 2*θ* < 40 (PANalytical, Netherlands). X-ray crystallographic intensity data were collected using a Bruker APEX-II CCD, Gemini Ultra CCD diffractometer equipped with a graphite monochromated Enhance (Mo) X-ray source (*λ* = 0.71073 Å).

### 2.2 High-pressure experiments

The crystal was placed in the hole of a T301 steel gasket, A 4 : 1 mixture of methanol and ethanol was used as a pressure transmitting medium (PTM). We conducted *in situ* steady-state PL measurements on an Ocean Optics QE65000 spectrometer in the reflection mode. Excitation source is the 355 nm line of a DPSS laser with a spot size of 20 mm. The diamond anvil cell (DAC) containing the sample was put on a Nikon fluorescence microscope to focus the laser on the sample. Optical photographs of the compressed *o*N-TPA samples were taken by an imaging camera (Canon EOS 5D Mark II) equipped on the fluorescence microscope. *In situ* transient-state PL was tested with a laser strobe spectro-fluorometer (produced by photon technology instruments) coupled to an inverted fluorescence microscope (Nikon Ti-U).

### 2.3 Lippert–Mataga model

The influence of solvent environment on the optical property of *o*N-TPA can be understood using the Lippert–Mataga equation:^19^1
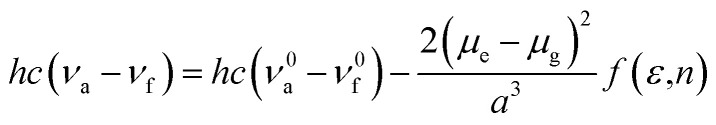
where *f* is the orientational polarizability of solvents, *ν*^0^_a_ − *ν*^0^_f_ corresponds to the Stokes-shift when *f* is zero. *μ*_e_ and *μ*_g_ are the dipole moments of the excited state and ground state, respectively, *a* is the solvent cavity (Onsager) radius, *ε* is the solvent dielectric, and *n* is the solvent refractive index.

### 2.4 Quantum chemical calculations

All the density functional theory (DFT) calculations were carried out using Gaussian 09 (version D.01) package on a Power Leader cluster. The excited state geometry was optimized by time-dependent density functional theory (TD-DFT) with the B3LYP functional at the basis set level of 6-31G(d,p). The emission properties were obtained using TD-M06-2X/6-31G(d,p) at the excited state geometries.

### 2.5 Synthesis and structural characterization of *o*N-TPA

Solution of cyano-TPA (1.8 g, 5 mmol) prepared according to the previous literatures^18*d*^ and 4-formylbenzonitrile (0.65 g, 6 mmol) in anhydrous EtOH (chromatographically pure, 30 ml) was treated with NaOCH_3_ (0.12 g, 2 mmol), stirred at room temperature for over 2 h, cooled to 0 °C, and filtered. The precipitate was repeatedly washed with EtOH to give the desired *o*N-TPA powders (2.2 g, 90%). *o*N-TPA: ^1^H NMR (500 MHz, DMSO-*d*_6_ see Fig. S1, ESI†) *δ* 8.76 (d, *J* = 4.0 Hz, 1H), 8.10 (s, 1H), 7.95–7.99 (m, 1H), 7.90 (d, *J* = 8.5 Hz, 2H), 7.82 (d, *J* = 8.5 Hz, 2H), *δ* 7.78 (d, *J* = 9.0 Hz, 1H), 7.69 (d, *J* = 9.0 Hz, 2H), 7.47–7.49 (m, 1H), 7.35 (t, *J* = 8.0 Hz, 4H), 7.05–7.11 (m, 8H); ^13^C NMR (500 MHz, DMSO-*d*_6_) *δ* = 154.4, 150.1, 147.5, 140.3, 136.8, 133.4, 129.4, 127.7, 127.1, 126.8, 124.7, 124.2, 123.5, 123.3, 117.4 and 114.6, MS(EI^+^): *m*/*z* 449.2.

## 3. Results and discussion

### 3.1 Solvatochromic effect

**Fig. 1:**
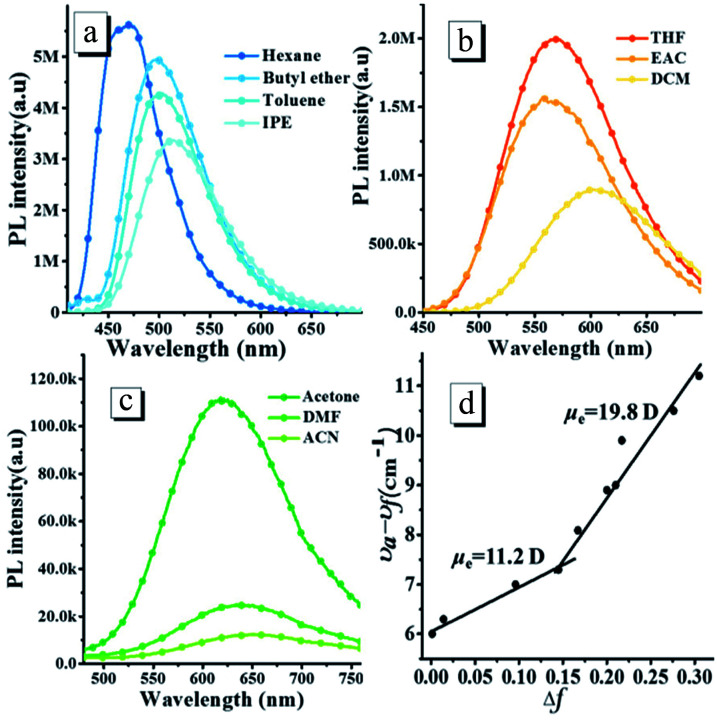
The fluorescence spectra (a–c) of *o*N-TPA measured in the different solvents with the increasing polarity (the orientational polarizability of solvent, Δ*f*, *n*–hexane: 0.0012; butyl ether: 0.096; toluene: 0.014; isopropyl ether (IPE): 0.145; tetrahydrofuran (THF):0.210; ethyl acetate (EAC): 0.200; dichloromethane (DCM): 0.217; *N*,*N*-dimethylformamide (DMF): 0.276; acetone: 0.284; and acetonitrile (ACN): 0.305), excitation at 400 nm, slit width 3; (d)the fitted linear correlation of the Stokes shift (*ν*_a_ − *ν*_f_) as a function of solvent polarity for *o*N-TPA in the low- and high-polarity regions.

The *o*N-TPA possesses a typical D–π–A structure, in which the triphenylamine group acts as the electron donor part against the acceptor of the cyano-group and pyridine. Since the photophysical properties of D–π–A molecules are dependent on the solvent polarity, the absorption and PL spectra of *o*N-TPA were measured in the different solvents (Fig. S2, ESI† and Fig. 1), and the detailed data shown in Table S1, ESI.† The absorption spectra of *o*N-TPA display the minor changes in both shape and position in the different solvents (Fig. S2†). In contrast, with the increase of solvent polarity, the emission peak demonstrates a gradual red-shift from 469 nm in hexane to 650 nm in acetonitrile, exhibiting a strong solvatochromic effect (Fig. 1a–c). At the same time, the vibronic fine structure disappears and emission band broadens from low polarity to high polarity solvents, which illustrates an obvious intra-molecular charge-transfer (ICT) character in excited state. In addition, a clear decreasing trend of the *η*_PL_ in the different solvents is observed, which is ascribed to the CT effect depending on solvent polarity.^19,20^

To evaluate the solvatochromic effect, the Lippert–Mataga model is applied to estimate the dipole moment (*μ*_e_) of the excited state. The plot of Stokes shift against orientation polarizability (Δ*f*) is shown in Fig. 1d. Notably, two linear relationships can be observed, indicating two different excited state properties in low and high polar solvents, respectively. The dipole moment, *μ*_e_, is calculated to be 19.8 D in high polarity solvents, which is near the typical CT compound DMABN with *μ*_e_ of 23 D.^21^ The rapid decrease of PL quantum yield from 59.6% in THF to 0.8% in acetonitrile also illustrates a CT-dominant excited state in high-polarity solvents. While in low-polarity solvents, the dipole moment is calculated to be 11.2 D, which is obviously smaller than that of CT state in high polarity solvents. However, the red shift of 39 nm from 0.0012 in hexane to 0.145 in isopropyl ether indicates that the CT character still exists for S_1_ state.^18^ Meanwhile, a vibration fine structure and relatively high *η*_PL_ in hexane demonstrate the existence of LE component. Thus, the S_1_ state in low-polarity solvents contains CT and LE components simultaneously. The *μ*_e_ of 11.2 D, which is smaller than DMABN but larger than the LE compound about 8 D,^21^ is ascribed to the HLCT state character. The LE component in HLCT state ensures a relatively high PL efficiency, and the CT component in the excited state results in a large Stokes shift from low polar solvents to high polar solvents, which is sensitive to the external stimuli.

### 3.2 Aggregation-induced enhanced emission

**Fig. 2:**
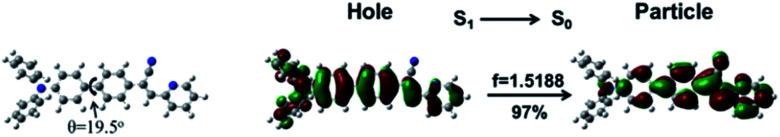
The S_1_→S_0_ NTO of *o*N-TPA. Herein, *f* represents the oscillator strength, and the weights of hole–particle are given for the S_1_→S_0_ excitation.

To better understand the nature of excited states, we calculated and analyzed the S_1_→S_0_ natural transition orbital (NTO)^22^ of *o*N-TPA as depicted in Fig. 2. For the S_1_ state, the hole is delocalized over the whole molecule, while the particle is mainly localized at the diphenylethylene group with a small part of benzene on the TPA group. The separated orbitals provide a CT component with large dipole moment. In addition, orbital overlaps on acceptor provide a LE component, which ensures a relatively high radiative-transition rate with the oscillator strength (*f*) of 1.5188. NTO analysis indicates the coexistence of LE and CT components. The twist angle between donor and acceptor is 19.5°. Benefiting from the proper twist angle, a balance of LE and CT component is achieved.

**Fig. 3:**
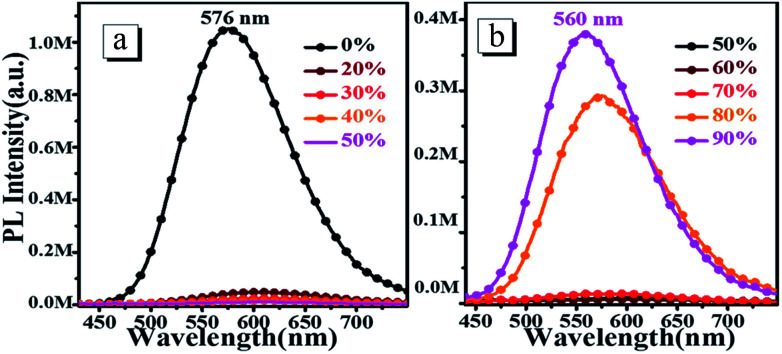
The steady-state fluorescence spectra of 10 μM *o*N-TPA in THF alone (0%) and THF–water mixture with water fractions from 20% to 90% (v/v).

To investigate the AIEE properties of *o*N-TPA, we measured the absorption and PL spectra in the water/THF mixture with different water fractions (Fig. S3, ESI† and Fig. 3). The fluorescence emissions demonstrate a slight red-shift and the intensities sharply decrease with gradual addition of water into the THF solution up to 70% (v/v), which can be ascribed to the enhanced ICT effect due to the increased solvent polarity. However, when the water fraction increases up to 80%, the aggregates appears, which can be verified by the lifted spectral tails from their absorption spectra. In addition, an obvious enhancement of the fluorescence intensity is observed because of the restriction in intramolecular rotations (RIR). As the water fractions reaches 90%, the fluorescence emission is further enhanced. Another reason for this AIEE behavior could be that the ICT process is effectually prevented, which is also helpful for the emission of light. Therefore, the emission maxima exhibit a blue-shift from 576 nm to 560 nm.

### 3.3 Water sensing

**Fig. 4:**
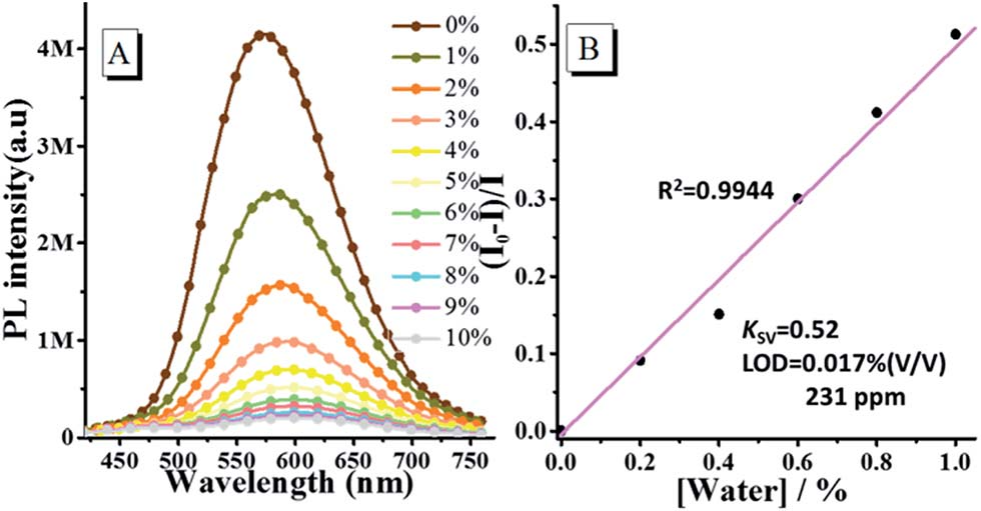
(A) PL spectra of 10 μM *o*N-TPA in THF/water mixture solvents with water fraction from 0 to 10% (v/v) (excitation at 400 nm, slit width 3). (B) The plot of (*I*_0_ − *I*)/*I* with water fractions from 0 to 10%. *I*_0_ and *I* represents the intensity in the absence and presence of water, respectively.

Based on the above results, the fluorescence emission of *o*N-TPA is highly sensitive to water, thus we further investigate its emission behaviour in THF by gradually increasing the content of water. Fig. 4a shows the fluorescence spectra of *o*N-TPA in THF with increasing the water fraction from 0 to 10% (v/v) in mixture solvents. In pure THF, *o*N-TPA exhibits a high PL efficiency of 59.6%, benefiting the full hybridization of LE and CT component. With the increase of water content, a significant decrease in the fluorescence intensity is observed, along with the red shift from 573 nm to 601 nm. It is ascribed to the synergistic effect of proton in water and ICT in mixture solvent. The fluorescence intensity is quenched by 61.9% when water content rises up to 3%. Especially, there is nearly no fluorescence emission when the water content reaches 7%. More importantly, a good linear relationship between fluorescent intensity (*I*_0_ − *I*)/*I* and water fraction is achieved when water fraction below 1% (Fig. S4, ESI†). The Stern–Volmer quenching constant (*K*_SV_) is 0.52 M^−1^, and the detection limit for water is 0.017% (231 ppm) in THF (Fig. 4b). Such a low detection limit indicates the high sensitivity of *o*N-TPA to water in THF. The results above suggest that *o*N-TPA can be used as a fast and effective PL probe to quantitatively detect low-level water in organic solvents.

### 3.4 Sensing properties toward protonic acids

#### 3.4.1 Response to pH in solvent

**Fig. 5:**
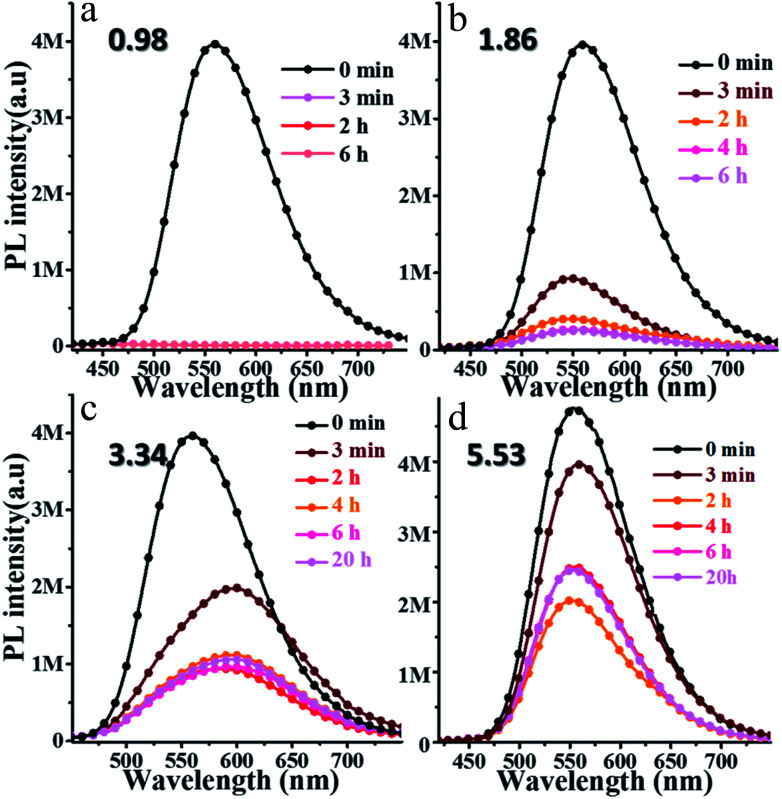
Time dependence of fluorescence spectra of 10 μM *o*N-TPA after adding the different TFA concentration to tune the pH values of solution to 0.98 (a), 1.86 (b), 3.34 (c), and 5.53 (d) in THF–water mixtures with volume ratios of 90% of water to THF (excitation at 400 nm, slit width 3).

*o*N-TPA may be used as a sensor for detecting H^+^, because the pyridine unit can bind with a proton to form a cation. To investigate its response to pH, the PL spectra of *o*N-TPA in THF–water mixtures with volume ratios of 1 : 9 at various pH were measured, and the pH was tuned by adding trifluoroacetic acid (TFA) concentration. As shown in Fig. 5a, after adding TFA for 3 minutes, the PL spectrum at pH 0.98 is almost a flat line parallel to the abscissa because of its transformation to the weakly emissive *o*N-TPAH^+^ under this acidic condition.^23^ When the pH increases to 1.86, there is still a weak fluorescence after 3 minutes (Fig. 5b). With the time getting longer, the *o*N-TPA molecule further undergoes protonation and its emission intensity gets weaker. As the pH further increases to 3.34 and 5.53 (Fig. 5c and d), the effect of proton quench reduces because of the progressive decrease in the population of *o*N-TPAH^+^ in solution. The extent of transformation of *o*N-TPA to *o*N-TPAH^+^ at the different pH values can also be followed by UV-vis absorption analysis (Fig. S5, ESI†). With the pH reduced from 4.52 to 0.96, the absorption peak at 374 nm nearly disappears and the band at 450 nm gradually increases, which should be ascribed to the formation of strong CT-state due to the increased electron withdrawing ability of protonated pyridine. At pH 4.52, a weak shoulder peak nearly disappears, showing that no protonation occurs at such a pH value.

#### 3.4.2 Response to HCl vapor in solid state

**Fig. 6:**
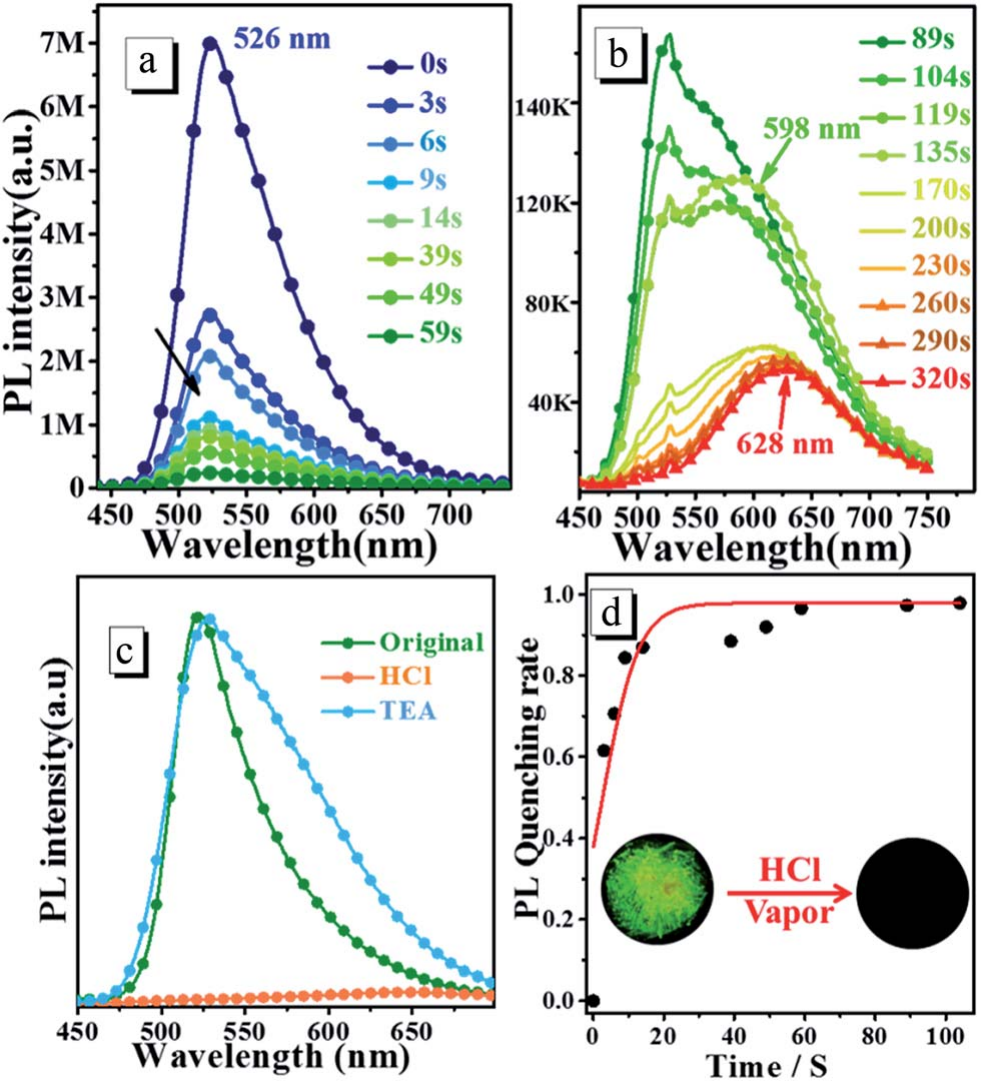
(a) and (b) Time dependence of steady-state fluorescence spectra of *o*N-TPA crystalline powders upon consecutive exposure to HCl vapors, (c) PL spectra of *o*N-TPA crystalline powders fumed by HCl and then TEA vapor, (d) time-course of PL quenching rate of *o*N-TPA original powders exposed to HCl vapors.

To determine the response towards HCl vapour in the solid state, the crystalline powder is exposed to HCl vapor for several minutes. Fig. 6 shows the time dependence of steady-state PL spectra of *o*N-TPA crystalline powders upon exposure to HCl vapour (60 ppm). The original crystalline powder exhibits a green emission at 526 nm and a relatively high *η*_PL_ of 77.2%, due to the AIEE effect. Upon exposed to HCl vapour, the fluorescence intensity significantly reduces due to the protonated pyridyl rings (see Fig. 6c). More than 85% of the fluorescence is quenched at 14 s. With the increasing exposing time in HCl vapour, another new peak at 598 nm appears as depicted in Fig. 6a and b. The quenching reaches equilibration about 170 s with the quenching rate of approximately 98%, that is, the population of *o*N-TPAH^+^ remains the same with the change of time. Finally, at 320 s there is a red emission band at 628 nm that should be ascribed to the strong CT emission from *o*N-TPAH^+^. Time-course of PL quenching rate is shown in Fig. 6d, the quenching rate is over 95% after 60 s. Furthermore, the reversibility experiment is carried out by exposing protonated crystalline powder to triethylamine (TEA) at room temperature. It is found that the emission maximum at 628 nm is quenched and the peak at 526 nm reappeared after a few minutes (see Fig. 6c). The recovered green fluorescence should be attributed to the deprotonation of pyridinium in *o*N-TPA-HCl by TEA to produce *o*N-TPA and NH_4_Cl. The crystalline powder after exposed to HCl vapor, significantly turns to red. In 365 nm UV light as shown in Fig. 6d, the green fluorescence disappears after protonation. This result demonstrates that *o*N-TPA could be used as a “naked-eye” and reversible sensing material for detecting HCl in practical applications.

**Fig. 7:**
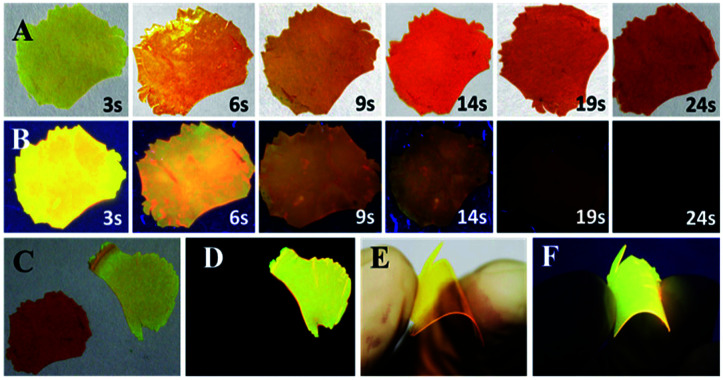
Photographs of the dye (*o*N-TPA, 5% wt/wt)-doped polymers (PMMA) film under 365 nm UV light (B, D and F) and natural light (A, C and E): (A and B) time dependence of the doped film upon consecutive exposure to HCl vapour (50 ppm), (C and D) the doped films before/after (final state,∼10 min) exposure to HCl vapor, (E and F) flexible thin-film.

The doped film is more favorable to application, because it possesses higher luminescence efficiency of 82.5% benefiting from the rigid environment and higher sensitivity to HCl vapor for its larger area to protonation. *o*N-TPA is dispersed into PMMA matrix using CHCl_3_ (5%, dye/PMMA, w/w) dried on neat quartz plate to obtain doped film. In the natural light, it presents yellow (see Fig. 7E), and it performances a bright yellow emission under 365 nm UV light (see Fig. 7F). Exposed to HCl vapor, the film rapidly changes from yellowish green to red under natural light (see Fig. 7A). The emission intensity under 365 nm UV light is quenched quickly with exposure time, fluorescence quenching more than 95% at 19 s, and no fluorescent signal can be measured after 24 s (see Fig. 7B). Photographs of original and protonated film-doped in natural light and 365 nm UV light are given out for comparison (see Fig. 7C and D). These results indicate that HCl is an efficient medium for quenching the fluorescence of *o*N-TPA, which can be used to quickly detect HCl vapor.

### 3.5 Mechanofluorochromic experiments

#### 3.5.1 Mechanical grinding experiments

**Fig. 8:**
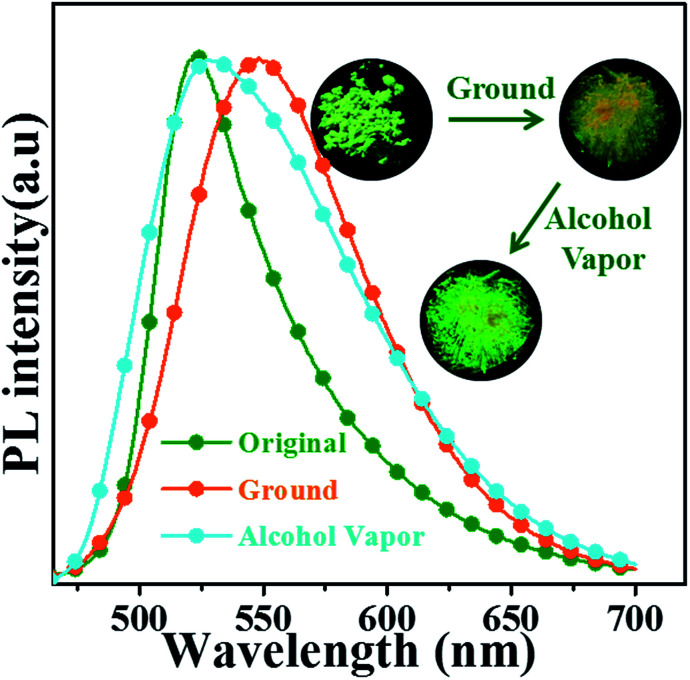
(a) Photographs and PL spectra of *o*N-TPA in the crystalline powders, the ground powders, and then exposed to alcohol vapour.

The *o*N-TPA is also sensitive to mechanical grinding and shows reversible fluorescence changes. As depicted in Fig. 8, the original crystalline powders exhibit a green fluorescence at 524 nm. Upon grinding, it displays a yellow emission with peak at 550 nm with the *η*_PL_ of 47.2%. The amorphous state shows a broadened PL spectrum and a red shift of 26 nm. It is further confirmed by identical their PXRD patterns (Fig. S6†). Compared to original sample, the ground powder displays significantly weakened and broadened peaks, indicating that the crystal structure is changed after grinding. Fuming with alcohol vapours, the fluorescence color recovers to green and the PXRD also recovers to original diffraction peaks, demonstrating the reversible switch between the crystalline powders and amorphous powders resulting in mechanochromic process. As depicted in Fig. S7,† the differential scanning calorimetry (DSC) thermogram of the ground powders revealed a clear endothermic peak at 106 °C, which was attributed to glass transition temperature (*T*_g_). However, the exothermic peak usually observed in other mechanochromic materials, did not appear.^20^ The result revealed that the phase transition (from amorphous to crystalline), was not achieved by heating treatments, which coincided with the experimental results.

#### 3.5.2 High pressure experiments

**Fig. 9:**
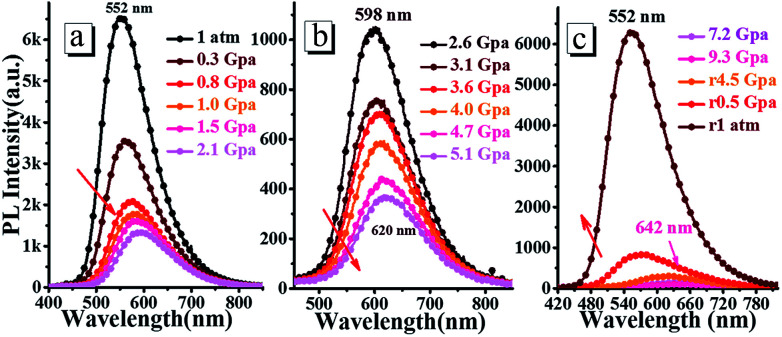
Steady-state fluorescence spectra of the doped film under the different hydrostatic pressure during compression (a and b) and decompression (c).

The high pressure experiments of *o*N-TPA were conducted by placing the doped film in the hole of a T301 steel gasket, using methanol and ethanol mixture (4 : 1) as a pressure-transmitting medium. As shown in Fig. 9, with the hydrostatic pressure gradually increased from 1 atm to 9.3 GPa, the fluorescence spectra demonstrate a remarkable red-shift from green (552 nm) to orange (598 nm), and further to red (642 nm). At the same time, the emission intensity is reduced continuously. In the nature light, the color of the doped film presents a change from green to red (Fig. S8†). Upon the pressure released to 1 atm, both color and fluorescence recover to the original state, indicating its good reversibility as a pressure sensor. The emissions can be switched between 552 and 642 nm (90 nm red-shift) during pressurizing–depressurizing process, which could be applied as piezochromic luminescent material.

## 4. Conclusions

In this work, we prepared a simple D–π–A structure multi-stimuli responsive fluorescence molecule of *o*N-TPA with AIEE effect. It exhibits obvious solvatochromic effect and its excited state is confirmed to be a HLCT state. Moreover, it can be used as a fast and effective PL probe to quantitatively detect low-level water in organic solvents, which is ascribed to the ICT effect with the increased mixture-solvents polarity. Because the pyridine unit can bind with a proton to form a cation, *o*N-TPA can also be used as a sensor for detecting protonic acids both in solvent for TFA and in solid for HCl vapor. Finally, it exhibits pronounced mechanochromic fluorescence properties, the reversibly emissions can be switched between 552 and 642 nm (90 nm red-shift) during pressurizing–depressurizing process. This work not only comprehensively demonstrates the stimuli-responsive fluorescence behaviors of *o*N-TPA, but also provides a D–π–A structure luminescent material possessing potential applications in detection and sensing with remarkable PL changes.

## Conflicts of interest

There are no conflicts to declare.

## Supplementary Material

RA-008-C8RA03598A-s001

## References

[cit1] Caruso M. M., Davis D. A., Shen Q., Odom S. A., Sottos N. R., White S. R., Moore J. S. (2009). Chem. Rev..

[cit2] Chung J. W., You Y., Huh H. S., An B. K., Yoon S. J., Kim S. H., Lee S. W., Park S. Y. (2009). J. Am. Chem. Soc..

[cit3] Luo X., Li J., Li C., Heng L., Dong Y. Q., Liu Z., Bo Z., Tang B. Z. (2011). Adv. Mater..

[cit4] Guo T., Deng Q., Fang G., Yun Y., Hu Y., Wang S. (2016). Biosens. Bioelectron..

[cit5] Zhang M., Zheng S., Ma L., Zhao M., Deng L., Yang L., Ma L. J. (2014). Spectrochim. Acta, Part A.

[cit6] Sheng L., Li M., Zhu S., Li H., Xi G., Li Y., Wang Y., Li Q., Liang S., Zhong K., Zhang. S. X.-A. (2014). Nat. Commun..

[cit7] Zhou T., Jia T., Zhao S., Guo J., Zhang H., Wang Y. (2011). Cryst. Growth Des..

[cit8] Luo J., Xie Z., Lam J. W., Cheng L., Chen H., Qiu C., Kwok H. S., Zhan X., Liu Y., Zhu D. (2001). Chem. Commun..

[cit9] Yao L., Zhang S., Wang R., Li W., Shen F., Yang B., Ma Y. (2014). Angew. Chem..

[cit10] Chen S., Liu J., Liu Y., Su H., Hong Y., Jim C. K., Kwok R. T., Zhao N., Qin W., Lam J. W., Wong K. S., Tang B. Z. (2012). Chem. Sci..

[cit11] Marín M. J., Galindo F., Thomas P., Wileman T., Russell D. A. (2013). Anal. Bioanal. Chem..

[cit12] Shi J., Zhang S., Zheng M., Deng Q., Zheng C., Li J., Huang F. (2017). Sens. Actuators, B.

[cit13] Feng C., Wang K., Xu Y., Liu L., Zou B., Lu P. (2016). Chem. Commun..

[cit14] Li W., Pan Y., Yao L., Liu H., Zhang S., Wang C., Shen F., Lu P., Yang B., Ma Y. (2014). Adv. Opt. Mater..

[cit15] Ramanan C., Smeigh A. L., Anthony J. E., Marks T. J., Wasielewski M. R. (2011). J. Am. Chem. Soc..

[cit16] Pron A., Gawrys P., Zagorska M., Djurado D., Demadrille R. (2010). Chem. Soc. Rev..

[cit17] Jiang Y., Wang Y., Hua J., Tang J., Li B., Qian S., Tian H. (2010). Chem. Commun..

[cit18] Li W., Pan Y., Xiao R., Peng Q., Zhang S., Ma D., Li F., Shen F., Wang Y., Yang B., Ma Y. (2014). Adv. Funct. Mater..

[cit19] Grabowski Z. R., Rotkiewicz K., Rettig W. (2003). Chem. Rev..

[cit20] Zhang Y., Qile M., Sun J., Xu M., Wang K., Cao F., Li W., Song Q., Zou B., Zhang C. (2016). J. Mater. Chem. C.

[cit21] Zhang S., Li W., Yao L., Pan Y., Shen F., Xiao R., Yang B., Ma Y. (2013). Chem. Commun..

[cit22] Martin R. L. (2003). J. Chem. Phys..

[cit23] Hariharan P. S., Pitchaimani J., Madhu V., Anthony S. P. (2017). Opt. Mater..

