# Supplementary material for: Stimuli-responsive fluorescence switching of cyanostilbene derivatives: ultrasensitive water, acidochromism and mechanochromism
Source: RSC Adv. 2018 Jun 21;8(40):22806–12. doi: 10.1039/c8ra03598a (PMC9081381; doi:10.1039/c8ra03598a)
Supplement: RA-008-C8RA03598A-s001 [file RA-008-C8RA03598A-s001.pdf]

Electronic Supplementary Material (ESI) for RSC Advances.
This journal is © The Royal Society of Chemistry 2018

Supporting Information

Bin Wang, [a,b] ChunYing Wei [a*]

[a] Key Laboratory of Chemical Biology and Molecular Engineering of Ministry of Education, Institute of Molecular Science, Shanxi University, Taiyuan 030006,P. R China. E-mail: weichuny@sxu.edu.cn

[b] School of Basic Medical Sciences, Shanxi Medical University, Taiyuan 030001,P. R. China. Tel:(+)86-351-7010699;

Contents

S-I Tables

S-II Figures

S-I Tables and figures

Table S1 Detailed absorption (λ_a) and emission peak (λ_f) positions of ***o*N-TPA** in the different solvents

solvents	$\Delta f\,(\varepsilon,n)$	***o*N-TPA**		
		λ_a (nm)	λ_f (nm)	ν_a-ν_f(cm^{-1})
Hexane	0.0012	370	474	6.0×10^3
Toluene	0.014	381	503	6.3×10^3
Butyl ether	0.096	370	499	7.0×10^3
Isopropyl ether	0.145	373	513	7.3×10^3
Ethyl ether	0.167	368	524	8.09×10^3
Ethyl acetate	0.200	375	561	8.9×10^3
Tetrahydrofuran	0.210	376	569	9.0×10^3
Dichloromethane	0.217	379	605	9.9×10^3
Dimethyl formamide	0.276	381	638	10.5×10^3
Acetonitrile	0.305	376	650	11.2×10^3

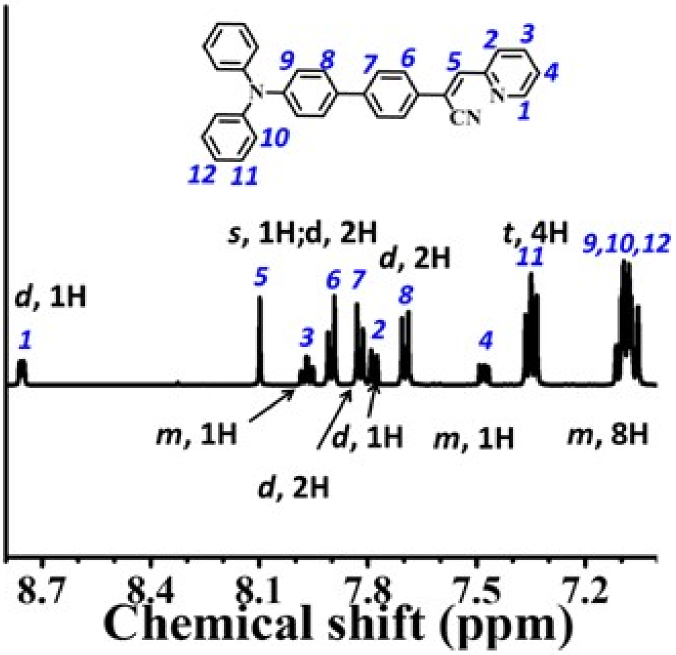

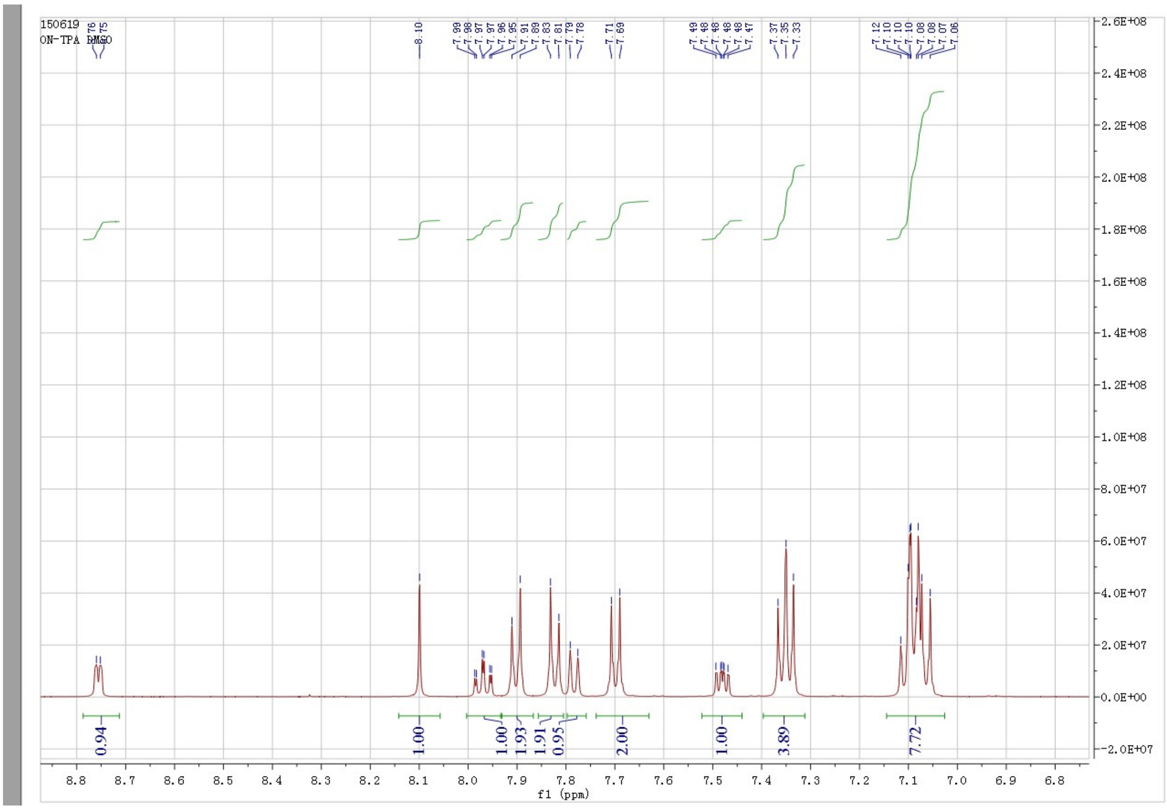

Figure S1 ^{1}**H NMR** of ***o*****N-TPA** in DMSO

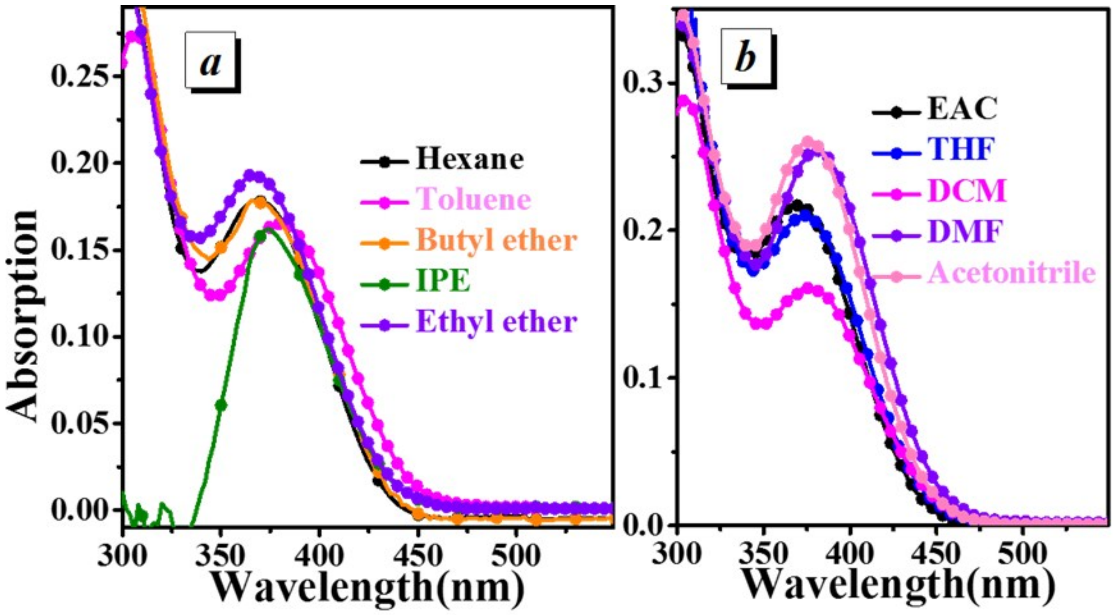

Figure S2 UV-vis absorption spectra of ***o*****N-TPA** measured in the different solvents with the increasing orientational polarizability (Δf). *n*–Hexane: 0.0012; toluene: 0.014; butyl ether: 0.096; isopropyl ether (IPE): 0.145; ethyl ether: 0.167; ethyl acetate (EA): 0.200; tetrahydrofuran (THF): 0.210; dimethyl formamide (DMF); and acetonitrile: 0.305.

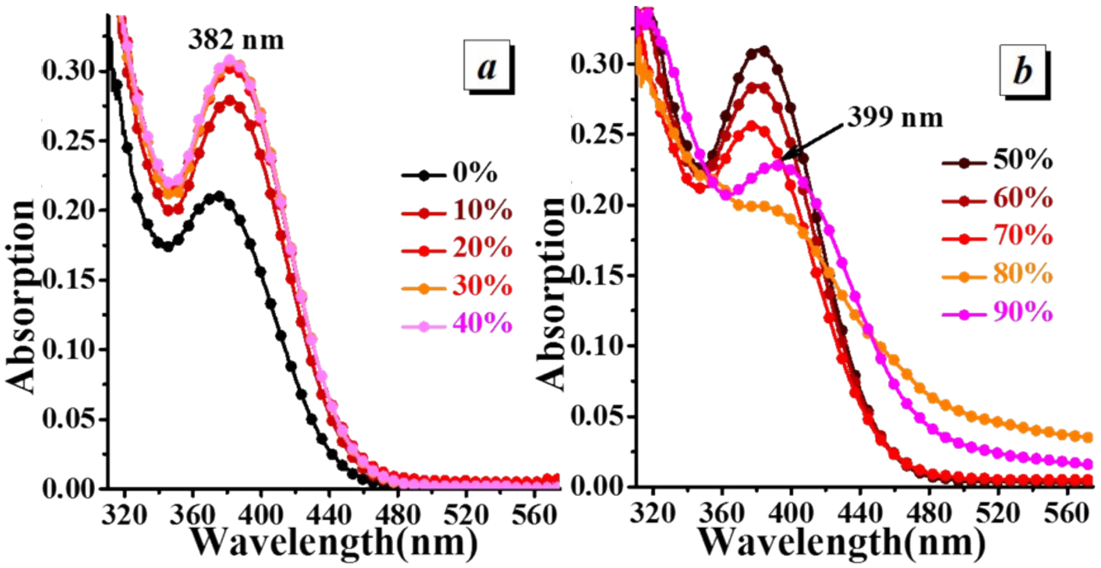

Figure S3 UV-vis absorption spectra of 10 μM ***o*N-TPA** in THF alone (0 %) and THF-water mixture with water fractions from 10 % to 90 % (v/v).

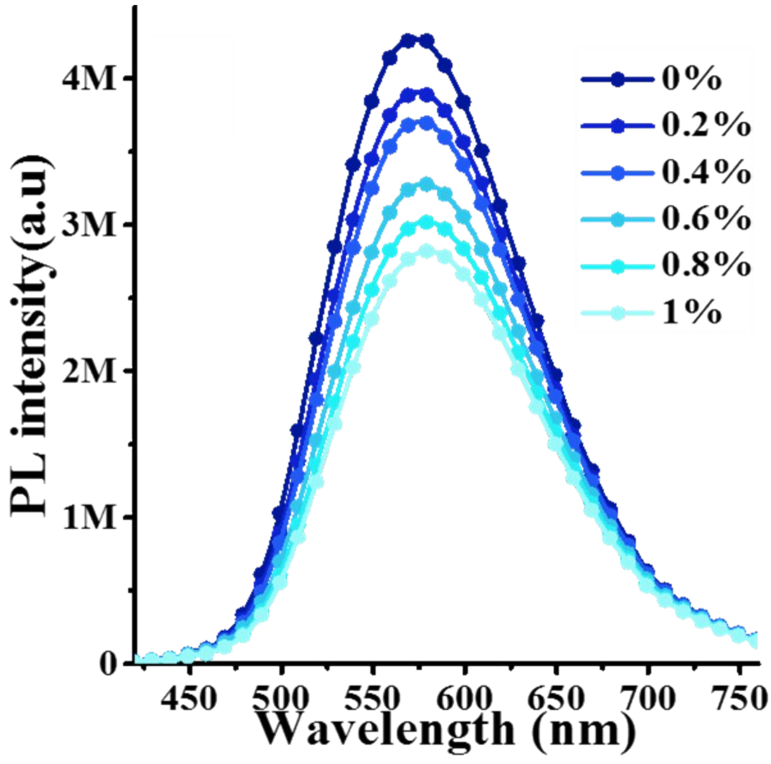

Figure S4 PL spectra change of 10 μM ***o*N-TPA** with the increasing water fraction from 0 to 1.0% (v/v)in THF (excitation at 410 nm, slit width 3).

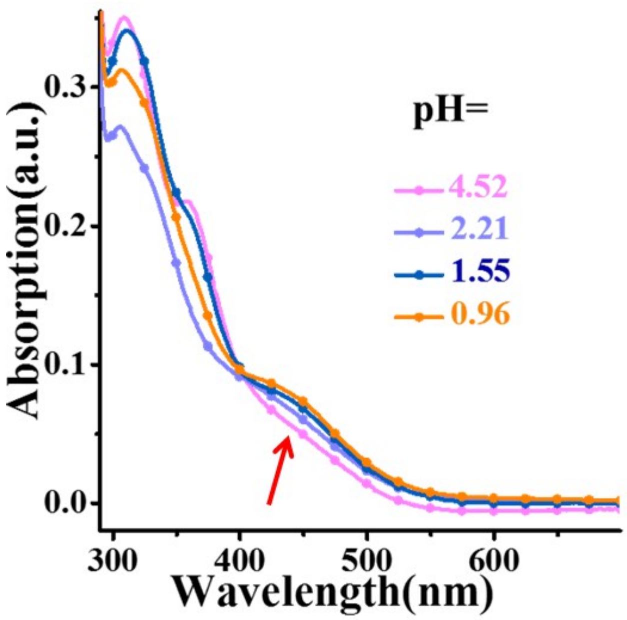

Fig.S5 Absorption spectra of 10 μM ***o*N-TPA** after adding the different concentration of TFA to tune the pH values of solution in THF-water mixtures with f_w=90%.

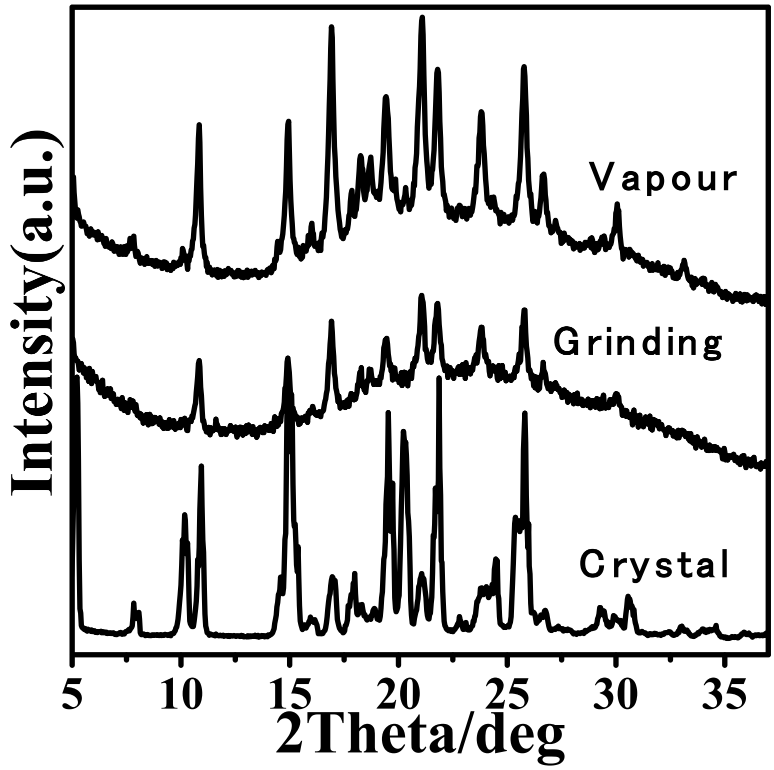

Fig. S6 Powder X-ray diffraction patterns of samples ***o*N-TPA** in the different states;

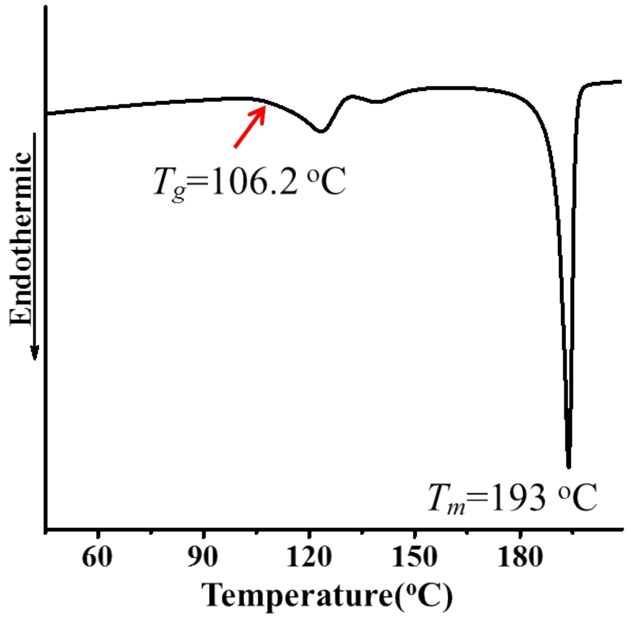

Fig. S7 the DSC curves (heating and cooling) of *o*N-TPA ground powders under a nitrogen atmosphere with heating and cooling rate of 10 °C min^{-1}

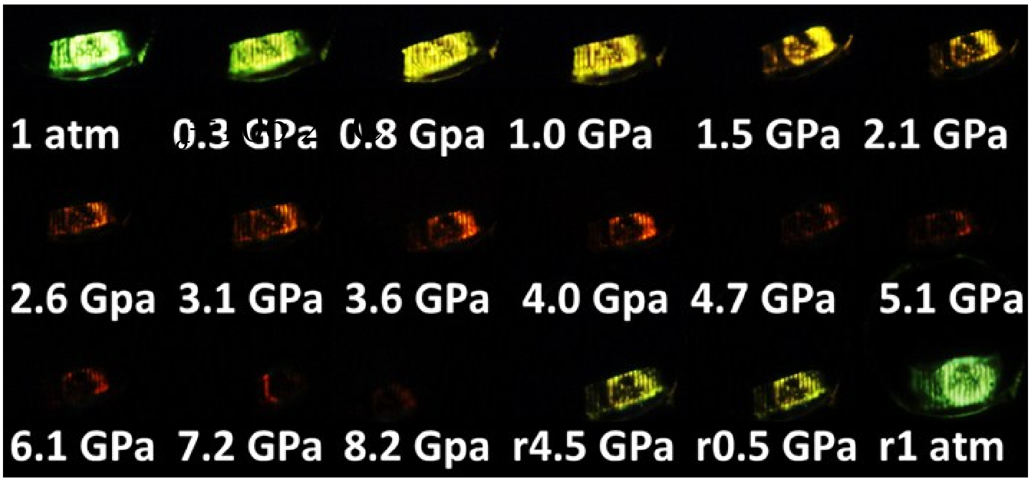

Fig. S8 fluorescence photographs of the Dye (***o*N-TPA**, 5% wt/wt)-doped polymers (**PMMA**) film under different hydrostatic pressure (from compression to decompression).
